# The diagnostic test performance of clinical point-of-care testing in relation to quantitative sensory testing for neurosensory injury among workers exposed to hand-arm vibration

**DOI:** 10.1093/joccuh/uiaf034

**Published:** 2025-06-13

**Authors:** Albin Stjernbrandt, Ingrid Liljelind, Eva Tekavec, Hans Pettersson

**Affiliations:** Department of Epidemiology and Global Health, Umeå University, Umeå, Sweden; Department of Epidemiology and Global Health, Umeå University, Umeå, Sweden; Division of Occupational and Environmental Medicine, Department of Laboratory Medicine, Lund University, Lund, Sweden; Department of Epidemiology and Global Health, Umeå University, Umeå, Sweden

**Keywords:** vibration, Sweden, peripheral nervous system diseases, screening, hand-arm vibration injury

## Abstract

**Objectives:**

Early detection of neurosensory injury among workers exposed to hand-arm vibration is crucial. The aim of our study was to evaluate the diagnostic test performance of clinical point-of-care testing using a tuning fork and temperature rollers in relation to vibrotactile and thermal quantitative sensory testing.

**Methods:**

We recruited 225 vibration-exposed workers who underwent clinical point-of-care testing using a Rydel Seiffer tuning fork and temperature rollers (25°C and 40°C) applied to the distal phalanges of the index and little fingers bilaterally. Quantitative sensory testing was conducted at the same locations. Sensitivity, specificity, and other measures of diagnostic test performance were calculated.

**Results:**

The study sample consisted of 208 men and 17 women with a median (IQR) age of 38 (26) years, and with a median (IQR) hand-arm vibration exposure duration of 12 (21) years. Using vibrotactile quantitative sensory testing as the reference method, the sensitivity for the Rydel Seiffer tuning fork to detect reduced perception of vibration ranged from 30.0% to 61.1%, depending on the tested finger. The corresponding values for specificity were 91.1% to 94.4%. The sensitivity of temperature roller discrimination in relation to warm detection thresholds ranged from 31.6% to 48.2%, and the specificity from 82.7% to 87.5%. The corresponding sensitivity of temperature roller discrimination in relation to cold detection thresholds ranged from 28.9% to 42.5%, and the specificity from 86.0% to 94.7%.

**Conclusions:**

The sensitivity of clinical point-of-care testing was rather low, indicating that quantitative sensory testing adds value to the diagnostic procedure.

## Teaser

Key points

- **What is already known on this topic:** Early detection of neurosensory hand-arm vibration injury is crucial for preventive measures. However, the diagnostic test performance of clinical point-of-care testing has not previously been evaluated in relation to quantitative sensory testing.
- **What this study adds:** The sensitivity of the Rydel Seiffer tuning fork was rather low, using vibrotactile quantitative sensory testing as the reference method, whereas the specificity was high. The same general pattern was found when evaluating temperature rollers in relation to thermal quantitative sensory testing. These findings suggest that quantitative sensory testing adds value to the diagnostic procedure.
- **How this study might affect research, practice, or policy:** The results of our study suggest that quantitative sensory testing could increase the detection rate of neurosensory injury among workers exposed to hand-arm vibration and should be implemented on a wider basis.

## 1. Introduction

Working with handheld vibrating tools means that energy is transferred from the tool to the contact surfaces of the hand, and prolonged exposure can cause hand-arm vibration (HAV) injuries.^1^ The most common HAV-related injuries are neurosensory impairment due to compromised function in afferent sensory nerves or mechanoreceptors, and vibration-induced white fingers (Raynaud phenomenon).^2^ Whereas the onset of the latter is usually obvious to the worker, neurosensory impairment is often more subtle as a clinical entity. Also, given an equal exposure, the neurosensory impairment has been reported to occur with a latency duration one-third that of vascular symptoms.^3^ Since there is no effective treatment for neurosensory impairment, early detection and appropriate preventive measures should be prioritized. According to current Swedish workplace legislation, workers exposed above the action value for HAV are to be offered periodical medical surveillance by the occupational health services (OHS) at employment and then every third year.^4^ This surveillance includes a screening questionnaire as well as clinical point-of-care testing of different modalities of sensory function in the hands.

Commonly used clinical point-of-care tools used by occupational physicians include monofilaments, 2-point discriminators, tuning forks, hot and cold temperature rollers, as well as grip and pinch strength dynamometers. Testing should ideally include both small and large fiber functions.^5^ Previous studies have shown that the tools used for clinical testing vary between OHS providers and that performing more tests increases the sensitivity of the medical surveillance program.^6^ Efforts have been made to standardize the testing procedure, and there are now national guidelines developed by the Swedish Trade Association for Occupational Health Services that regulate how HAV-exposed workers should be periodically examined.^5^ Moreover, some larger OHS as well as all the Occupational and Environmental Medicine Clinics at the university hospitals in Sweden employ quantitative sensory testing (QST) to diagnose neurosensory HAV injury. This is a psychophysical testing procedure where detection thresholds for different kinds of stimuli are determined. The most commonly investigated modalities include vibration and thermal perception thresholds,^7^^,^^8^ and there are several brands of test equipment that are used internationally, not only for diagnosing HAV injury but also for peripheral nerve impairment related to diabetes and chemotherapy.^9-11^ However, there is a lack of knowledge about the agreement between clinical point-of-care testing and QST. Further, the potential added diagnostic value of determining vibration and thermal perception thresholds in addition to clinical point-of-care testing has not been previously evaluated.

The aim of our study was therefore to evaluate the diagnostic test performance of clinical point-of-care testing in relation to QST for neurosensory injury among workers exposed to HAV.

## 2. Materials and methods

### 2.1. Study design

We recruited HAV-exposed construction workers, mechanics, and welders living in the counties of Västerbotten or Västernorrland in northern Sweden. Commonly used tools included screwdrivers, impact wrenches, circular and reciprocating saws, and grinders. In addition, we also included patients referred to the Occupational and Environmental Medicine Clinic at the University Hospital of Umeå due to suspected HAV injury of any sort. This clinic accepts referrals of patients from all counties in northern Sweden. Recruitment and data collection were conducted between August 2021 and February 2024, and a thorough description has previously been published.^12^ In brief, workers were asked to complete a written survey to provide data on age, sex, height, weight, tobacco habits, and HAV exposure. They then underwent standard clinical point-of-care testing, either performed by 2 of the authors (I.L. and H.P.) or by physicians at the clinic. Vibrotactile and thermal QST was performed by 1 of the authors (H.P.) or by staff members at the clinic. All testing adhered to strict examination protocols that were developed for the purpose of this study and adhered to national guidelines of the Swedish Trade Association for Occupational Health Services.

### 2.2. Clinical point-of-care testing

The testing was performed in a quiet room with normal indoor temperature (20°C to 22°C), with the worker sitting comfortably in a chair with the hands placed on an examining table. The finger skin temperature was registered before examination using an infrared thermometer (Testo 845; Testo, Alton, Hampshire, UK), and if below 28°C, the hands were warmed using hot water. All workers were instructed to avoid nicotine, caffeine, and strenuous physical activities for 2 hours before testing, and to avoid HAV exposure for the entire preceding day. Clinical neurosensory testing was performed on the distal volar aspects of the index and little fingers of both hands. Perception of vibration was tested using a Rydel Seiffer tuning fork, which has a frequency of 128 Hz but is dampened to 64 Hz using scaled metal blocks attached to the prongs. When the tuning fork is set in motion, an illusion of 2 triangles appears on each prong, and the intersection of these triangles gradually moves up a scale ranging from 0 (maximum vibration) to 8 (no vibration). The disappearance threshold (ie, when the subject no longer can feel the vibration) was recorded, and a mean threshold below 6.0 based on 3 attempts was considered abnormal.^13^ Perception of temperature was tested using 25°C and 40°C temperature rollers (Somedic SenseLab Rolltemp®, Sösdala, Sweden), where being unable to distinguish between the warm and cold temperature rollers at the distal phalanx in at least 1 of 3 attempts was considered a sign of impaired temperature discrimination. These 2 clinical point-of-care tests were selected since they are recommended in the national guidelines of the Swedish Trade Association for Occupational Health Services and test both myelinated and unmyelinated afferent fibers.

### 2.3. Vibrotactile and thermal QST

Participants underwent QST, where vibration perception thresholds (VPTs) of the index and little fingers of both hands were determined using the Vibro-Sense Meter II (VibroSense Dynamics, Malmö, Sweden). The participant placed the pulp of the tested finger on a vibrating pin on the device and pressed a button with the other hand when the vibration stimulus was perceived. This machine measures VPT for 7 frequencies (8, 16, 32, 64, 125, 250, and 500 Hz) and calculates an average threshold for all frequencies that is expressed as the deviation from the mean value of an age- and sex-matched reference population (ie, average *z* score (*z*_avg_)].^7^ A *z*_avg_ > 1.7 was considered abnormal. A subset of participants (*n* = 8) was examined using the first generation of the Vibro-Sense Meter, which calculates sensibility index (SI),^14^ and this was converted to the average *z* score using the formula: *z*_avg_ = 7.5 − 6.8 × SI.

Warm detection thresholds (WDTs) and cold detection thresholds (CDTs) were established by the method-of-limits^15^ using a SenseLab Modular Sensory Analyser coupled to a 25 × 50 mm thermode (Somedic SenseLab AB, Sösdala, Sweden).^16^ The participant placed the pulp of the tested finger on the thermode and pressed a button with the other hand when perceiving thermal stimuli, which triggered the read-out of thermode temperature and return to starting temperature. The machine was programmed to perform 5 consecutive cold stimuli followed by 5 warm stimuli with random inter-stimuli intervals of 4 to 6 seconds, using a starting temperature of 32.0°C ± 0.1°C, change rate of 1.0°C per second during stimuli, and return rate of 3.0°C per second. The mean value of 5 stimuli was calculated after correcting for any unintended pressing of the button. An average WDT ≥ 38.0°C and CDT ≤ 28.0°C was considered abnormal. All the results were individually assessed by the study physician (A.S.) and participants with abnormal findings were offered a follow-up visit to the clinic. The protocol for our study was approved by the Swedish Ethical Review Authority (DNR 2021-01463).

### 2.4. Statistical methods

The study sample size was dimensioned to reach sufficient precision when estimating sensitivity and specificity, assuming a 40% prevalence of abnormal findings during clinical testing in at least 1 sensory modality. Using the Buderer equation,^17^ 202 participants would be required to reach a target margin of error of 10% (ie, a 95% CI of 70% ± 10%). Numerical data violated assumptions of normal distribution and were therefore presented as median values with interquartile ranges (IQRs). Categorial data were presented as numbers (*n*) and valid percentage. Missing data were excluded from analyses. Spearman rank correlation coefficient (*r*_s_) was used to test the correlation between age and exposure duration. The Mann-Whitney *U* test and Fisher exact test were used to test differences between independent samples, whereas the Wilcoxon signed rank test and McNemar test were used for dependent samples (eg, different fingers on the same hand). Sensitivity, specificity, positive and negative predictive values, positive and negative likelihood ratios, and diagnostic odds ratios (ORs) were calculated based on cross-tabulation of outcomes of clinical point-of-care testing in relation to QST, where the latter was considered the reference method. We calculated 95% CIs for diagnostic test performance assuming a binominal distribution of data.

## 3. Results

### 3.1. Participants

Our study recruited 225 workers with a median (IQR) age of 38 (26) years, of whom 17 (7.6%) were female (Table 1). The median (IQR) self-reported HAV exposure duration was 12 (21) years, and 69 (32.5%) had been exposed for more than 20 years. The correlation between age and exposure duration was high (*r*_s_ = 0.81, *P* < .001). Regarding previous injuries and diseases, there were 62 subjects (27.6%) who had previous forearm or hand injuries (eg, lacerations or crush injuries), 21 subjects (9.3%) with previous neurological conditions (eg, carpal tunnel syndrome, nerve injury, or multiple sclerosis), 15 (6.7%) with diabetes mellitus, and 5 (2.6%) with previous heart disease (angina pectoris, myocardial infarction, congestive heart failure, or pacemaker dependency). There were 2 subjects (0.9%) who reported having had thyroid disease but only 1 used levothyroxine supplementation. Further, there were 3 (1.3%) with vitamin B deficiency and 1 (0.4%) who reported alcohol dependency. Regarding medications that could influence the peripheral nervous system, 12 (5.3%) reported using lipid-lowering drugs (simvastatin, atorvastatin, and rosuvastatin), 2 (0.9%) immunosuppressive treatment (rituximab, infliximab, and methotrexate), 2 (0.9%) antiepileptic drugs (topiramate and gabapentin), 2 (0.9%) previous chemotherapy, and 1 (0.4%) systemic acne treatment (isotretinoin). Regarding hereditary neurological conditions, 2 (0.9%) reported having relatives with Parkinson disease, 2 (0.9%) with familial amyloid polyneuropathy, and 1 (0.4%) with essential tremor. All recruits participated in clinical testing and vibrotactile QST. Due to technical difficulties, only 166 participants (73.8%) underwent thermal QST. Those who completed thermal QST were slightly older (median [IQR] age 39 [26] years vs 34 [22] years, *P* = .011) but there was no difference with regards to female sex (7.8% vs 6.8%, *P* = .525) nor in the proportion of abnormal findings when tested using temperature rollers (*P* = .232 to .743, depending on the tested finger).

**Table 1:** Descriptive data for study participants.

**Variable**	**Categories**	**Men**	**Women**
		***n* (%)**	***n* (%)**
Age group, y	19-35	89 (42.8)	9 (52.9)
	36-51	54 (26.0)	4 (23.5)
	52-67	65 (31.3)	4 (23.5)
			
Body mass index, kg/m^2^	18.5-25.0	55 (26.7)	11 (64.7)
	>25-30	106 (51.5)	5 (29.4)
	>30	45 (21.8)	1 (5.9)
			
Smoking	Never	143 (68.8)	9 (56.3)
	Former	55 (26.4)	7 (43.8)
	Current	10 (4.8)	0 (0.0)
			
Snuff use	Never	94 (45.6)	7 (41.2)
	Former	23 (11.2)	3 (17.6)
	Current	89 (43.2)	7 (41.2)
			
Hand-arm vibration exposure duration, y	<5	31 (15.8)	7 (43.8)
	5-20	98 (50.0)	7 (43.8)
	>20	67 (34.2)	2 (12.4)

### 3.2. Clinical point-of-care testing

The median (IQR) disappearance threshold for the Rydel Seiffer tuning fork (based on the mean value of 3 attempts) was 7.50 (1.29) for the right index finger and 7.00 (1.29) for the right little finger (*P* < .001). For the left hand, the median (IQR) was 7.33 (1.17) for the left index finger, and 7.00 (1.33) for the left little finger (*P* < .001). Detailed results are presented in Table 2. Abnormal disappearance thresholds (ie, a mean value below 6.0 based on 3 attempts) were found for 9.7% to 22.3% of the participants, depending on the tested finger. More participants had abnormal disappearance thresholds in the little fingers than the index fingers (right hand 22.3% vs 9.7%; left hand 17.3% vs 13.1%). An inability to discriminate between the warm and cold temperature rollers at the distal phalanges was found for 18.3% to 34.3% of the participants, depending on the tested finger. More participants had abnormal thermal discriminative capacity in the little fingers compared with the index fingers (right hand 34.3% vs 20.1%, *P* < .001; left hand 29.5% vs 18.3%, *P* < .001).

**Table 2:** Results from clinical point-of-care and quantitative sensory testing for vibrotactile and thermal sensibility.

		**Right**		**Left**	
**Test**	**Categories**	**Index finger** ***n* (%)**	**Little finger** ***n* (%)**	**Index finger** ***n* (%)**	**Little finger** ***n* (%)**
Rydel Seiffer tuning fork (disappearance threshold)	>7	121 (61.4)	71 (36.0)	120 (60.6)	89 (45.2)
	>6-7	57 (28.9)	82 (41.6)	52 (26.3)	74 (37.6)
	>5-6	12 (6.1)	29 (14.7)	17 (8.6)	22 (11.2)
	≤5	7 (3.6)	15 (7.6)	9 (4.5)	12 (6.1)
					
Vibrotactile quantitative sensory testing: vibration perception threshold (*z*_avg_)	<1.0	169 (75.1)	149 (66.2)	172 (76.4)	148 (66.1)
	1.0-1.7	31 (13.8)	36 (16.0)	31 (13.8)	42 (18.8)
	>1.7	9 (4.0)	14 (6.2)	8 (3.6)	12 (5.4)
	≥2.0	16 (7.1)	26 (11.6)	14 (6.2)	22 (9.8)
					
40°C/25°C temperature rollers (most distal point of discrimination)	Distal phalanx	175 (79.9)	140 (65.7)	178 (81.7)	155 (70.5)
	Middle phalanx	31 (14.2)	54 (25.4)	31 (14.2)	43 (19.5)
	Proximal phalanx	11 (5.0)	16 (7.5)	9 (4.1)	16 (7.3)
	Palm or proximally	2 (0.9)	3 (1.4)	—	6 (2.7)
					
Thermal quantitative sensory testing: warm detection threshold, °C	<38.0	106 (63.9)	78 (47.0)	98 (59.4)	65 (39.6)
	38.0-42.0	41 (24.7)	56 (33.7)	42 (25.5)	60 (36.6)
	>42.0	19 (11.4)	32 (19.3)	25 (15.2)	39 (23.8)
					
Thermal quantitative sensory testing: cold detection threshold, °C	>28.0	74 (44.6)	40 (24.1)	65 (39.4)	50 (30.3)
	23.0-28.0	78 (47.0)	96 (57.8)	81 (49.1)	79 (47.9)
	<23.0	14 (8.4)	30 (18.1)	19 (11.5)	36 (21.8)

**Figure 1:**
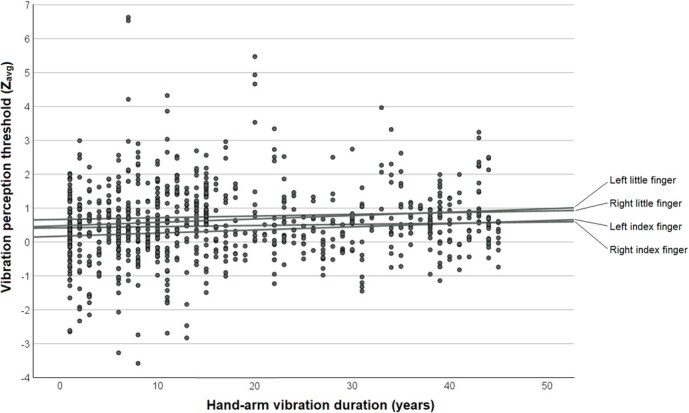
Scatter plot of vibration perception thresholds (average *z* values) from vibrotactile quantitative sensory testing by years of hand-arm vibration exposure duration. The *z* values represent averages of all the 7 tested frequencies (8, 16, 32, 64, 125, 250, and 500 Hz) and are age- and sex-matched. The linear fit lines represent the 4 fingers.

**Figure 2:**
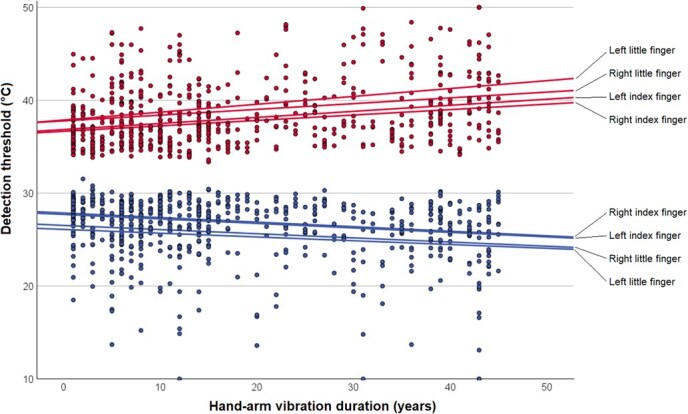
Scatter plot of warm detection thresholds (upper, red) and cold detection thresholds (lower, blue) from thermal quantitative sensory testing by years of hand-arm vibration exposure duration. The linear fit lines represent the 4 fingers.

### 3.3. Vibrotactile and thermal QST

Regarding vibrotactile QST, the median (IQR) *z*_avg_ was 0.4 (1.1) for the right index finger, 0.7 (1.2) for the right little finger, 0.3 (1.2) for the left index finger, and 0.6 (1.3) for the left little finger. Detailed results are presented in Table 2. The difference in VPT (*z*_avg_) between the index and little finger was statistically significant for both the right and left hand (*P* < .001 for both). Abnormal VPT (*z*_avg_ > 1.7) was found in 9.8% to 17.8% of participants (Table 2), depending on the finger, with similar predominance in the little fingers compared with the index fingers. There was a general trend where the VPT (*z*_avg_) increased with more years of HAV exposure (Figure 1). Regarding thermal QST, the median (IQR) WDT was 37.1°C (3.5°C) for the right index finger, 38.1°C (4.8°C) for the right little finger, 37.3°C (4.1°C) for the left index finger, and 39.0°C (5.4°C) for the left little finger. The WDT was significantly higher in the little fingers compared with the index fingers for both hands (*P* < .001 for both), whereas the CDT was lower in the little fingers compared with the index fingers bilaterally (*P* < .001 for both). Abnormal WDT (≥38.0°C) was found for 36.1% to 60.4% of participants, depending on finger. For abnormal CDT (≤28.0°C), the corresponding interval was 55.4% to 75.9%. The WDT and CDT diverged with more years of HAV exposure (Figure 2).

### 3.4. Agreement between clinical point-of-care and QST

Using vibrotactile QST as the reference method, the sensitivity for the Rydel Seiffer tuning fork to detect reduced perception of vibration ranged from 30.0% to 61.1%, depending on the tested finger. The corresponding values for specificity were 91.1% to 94.4%. The sensitivity of temperature roller discrimination in relation to WDT ranged from 31.6% to 48.2%, and the specificity from 82.7% to 87.5%. The corresponding sensitivity of temperature roller discrimination in relation to CDT ranged from 28.9% to 42.5%, and the specificity from 86.0% to 94.7%. Positive and negative predictive values, positive and negative likelihood ratios, and diagnostic ORs for the clinical point-of-care tests are presented in Table 3.

### 3.5. Sensitivity analyses

The analyses of diagnostic test performance were reiterated using different thresholds for abnormality for vibrotactile and thermal QST, to determine how a wider definition of normality would influence the diagnostic precision of clinical point-of-care tools (Table S1). For VPT, abnormality was defined as *z*_avg_ > 2.0, which would indicate “severe pathology” according to the manufacturer of the instrument. For thermal QST, a WDT >42.0°C and CDT <23.0°C was selected to define abnormality since these cut-offs are currently used at several occupational and environmental medicine clinics in Sweden. By using these wider thresholds, fewer subjects were defined as having abnormal findings according to QST (Table S1). However, the sensitivity of the Rydell Seiffer tuning fork was still rather low (45.5% to 63.6%), and the same was true for temperature rollers in relation to WDT (36.1% to 64.5%) and CDT (51.6% to 58.3%). Conversely, the specificity of clinical point-of-care testing was slightly reduced.

## 4. Discussion

### 4.1. Key results

When evaluating the diagnostic test performance of the Rydel Seiffer tuning fork using vibrotactile QST as the reference method, the sensitivity was rather low whereas the specificity was high. The same general pattern was found when evaluating temperature rollers in relation to thermal QST. These findings suggest that vibrotactile and thermal QST add value to the diagnostic procedure. However, the high specificity of the clinical point-of-care tools indicates that there were few false positive findings associated with these tests.

**Table 3:** Diagnostic test performance of the Rydel Seiffer tuning fork and temperature rollers, using vibrotactile and thermal quantitative sensory testing as reference methods.

**Clinical test**	**Anatomical location**	**Abnormal finding**	**Quantitative sensory testing**	**Abnormal finding**	**Sensitivity (%)**	**Specificity (%)**	**Positive predictive value (%)**	**Negative predictive value (%)**	**Positive likelihood ratio**	**Negative likelihood ratio**	**Diagnostic odds ratio**
		***n* (%)**		***n* (%)**	**Estimate (95% CI)**	**Estimate (95% CI)**	**Estimate (95% CI)**	**Estimate (95% CI)**	**Estimate (95% CI)**	**Estimate (95% CI)**	**Estimate**
Rydel Seiffer tuning fork	Right index finger	16 (8.1)	Vibration perception threshold (*z*_avg_) >1.7	25 (11.1)	30.0 (14.5-51.9)	94.4 (89.9-96.9)	37.5 (18.5-61.4)	92.3 (87.4-95.3)	5.4 (2.2-13.1)	0.7 (0.6-1.0)	7.2
	Right little finger	33 (16.8)	Vibration perception threshold (*z*_avg_) >1.7	40 (17.8)	41.2 (26.4-57.8)	91.1 (85.7-94.6)	50.0 (32.6-67.4)	87.8 (81.9-92.0)	4.6 (2.5-8.8)	0.7 (0.5-0.9)	7.1
	Left index finger	22 (11.1)	Vibration perception threshold (*z*_avg_) >1.7	22 (9.8)	61.1 (38.6-79.7)	93.9 (89.4-96.6)	50.0 (30.7-69.3)	96.0 (92.0-98.1)	10.0 (5.1-19.8)	0.4 (0.2-0.7)	24.2
	Left little finger	27 (13.7)	Vibration perception threshold (*z*_avg_) >1.7	34 (15.2)	46.4 (29.5-64.2)	91.7 (86.6-95.0)	48.1 (30.7-66.0)	91.2 (86.0-94.6)	5.6 (3.0-10.6)	0.6 (0.4-0.8)	9.6
Temperature rollers	Right index finger	44 (20.1)	Warm detection threshold (≥38.0°C)	60 (36.1)	31.6 (21.0-44.5)	87.5 (79.8-92.5)	58.1 (40.8-73.6)	70.0 (61.6-77.2)	2.5 (1.3-4.8)	0.8 (0.7-1.0)	3.2
			Cold detection threshold (≤28.0°C)	92 (55.4)	30.3 (21.8-40.5)	94.4 (86.6-97.8)	87.1 (71.1-94.9)	52.3 (43.8-60.7)	5.4 (2.0-14.9)	0.7 (0.6-0.9)	7.3
	Right little finger	73 (34.3)	Warm detection threshold (≥38.0°C)	88 (53.0)	48.2 (37.8-58.8)	82.7 (72.6-89.6)	75.5 (62.4-85.1)	59.0 (49.5-68.0)	2.8 (1.6-4.8)	0.6 (0.5-0.8)	4.5
			Cold detection threshold (≤28.0°C)	126 (75.9)	42.5 (34.0-51.4)	94.7 (82.7-98.5)	96.2 (87.2-99.0)	34.3 (25.9-43.8)	8.0 (2.1-31.6)	0.6 (0.5-0.7)	13.2
	Left index finger	40 (18.3)	Warm detection threshold (≥38.0°C)	67 (40.6)	32.3 (22.2-44.4)	87.5 (79.4-92.7)	63.6 (46.6-77.8)	65.6 (57.0-73.3)	2.6 (1.4-4.9)	0.8 (0.6-0.9)	3.3
			Cold detection threshold (≤28.0°C)	100 (60.6)	28.9 (20.8-38.6)	92.2 (83.0-96.6)	84.8 (69.1-93.3)	46.1 (37.7-54.7)	3.7 (1.5-9.1)	0.8 (0.7-0.9)	4.8
	Left little finger	65 (29.5)	Warm detection threshold (≥38.0°C)	99 (60.4)	33.7 (25.0-43.7)	82.8 (71.8-90.1)	74.4 (59.8-85.1)	45.7 (36.9-54.7)	2.0 (1.1-3.6)	0.8 (0.7-1.0)	2.5
			Cold detection threshold (≤28.0°C)	115 (69.7)	33.6 (25.5-42.9)	86.0 (73.8-93.0)	84.1 (70.6-92.1)	37.1 (28.8-46.1)	2.4 (1.2-5.0)	0.8 (0.7-0.9)	3.1

### 4.2. Interpretation

It is challenging to define reduced neurosensory function in the hands, both from a clinical and technical standpoint. In our study, the normative values for vibrotactile QST on the distal phalanges of the fingers followed the recommendations of the manufacturer of the instrument, which in turn are based on Swedish reference populations where VPTs have been determined in both healthy adults and children.^7^^,^^18^ In the reference material on adults, relevant to our study, vibrotactile perception deteriorated with age but was unaffected by sex. In our study, age was strongly associated with HAV exposure duration, and both natural aging and HAV exposure are likely to lead to a deterioration in vibrotactile perception.^2^ This was illustrated by the increasing *z*_avg_ with HAV exposure duration (Figure 1). Moreover, there were too few women in our study to be able to determine any potential effect of sex. When it comes to thermal QST, there are several previous reference materials, but some of these populations were exposed to factors that could negatively affect the WDT and CDT. Rolke et al^8^ investigated 180 healthy subjects and based on the reported 95% CI, a WDT <36.2°C to 38.1°C could be considered normal, the range dependent on both sex and age. The corresponding threshold for CDT was >27.7°C to 28.9°C. Burström et al^19^ investigated 251 Arctic open-pit miners where exposure to HAV and cold climate was common, and based on the 95% CI, a WDT <37.1°C to 37.5°C and CDT >28.1°C to 28.3°C could be considered normal, depending on the tested finger and sex. There are also other previous reports of WDT and CDT in the hands of HAV-exposed workers and controls,^20^^,^^21^ cold-exposed military conscripts,^22^ obese patients and controls (*n* = 41), as well as healthy subjects.^23^ Interestingly, Lundström et al^24^ indicated that age had a significant impact on WDT and CDT in HAV-exposed workers. In our study, we defined abnormality based on WDT and CDT that resembled the results of Rolke et al^8^ and Burström et al.^19^ However, we also performed a sensitivity analysis with a wider definition of normality than is currently used at several Occupational and Environmental Medicine Clinics in Sweden. In these analyses we used a *z*_avg_ > 2.0, WDT >42.0°C, and CDT <23.0°C as cut-offs for abnormal findings. As expected, this resulted in higher sensitivity and lower specificity for clinical point-of-care testing. However, the sensitivity was still low enough for clinical point-of-care testing to motivate additional testing using QST. There were also some differences in the proportions of abnormal findings between CDT and WDT, where slightly more participants had abnormal thresholds for cold stimuli. This may partly be explained by the fact that the method-of-limits uses a fixed starting temperature of 32.0°C, which is not centered in the reference interval and is higher than the finger skin temperature of many workers, although we actively heated hands with a temperature less than 28.0°C. Since the ability to discriminate temperature change is dependent on the actual finger skin temperature, the fixed starting temperature may have introduced a skewness in detection thresholds. Alternatively, the asymmetry between abnormal CDT and WDT may reflect functional differences in afferent nerve fibers of the Aδ- and C-types.

Apart from sensitivity and specificity, we also calculated other measures of diagnostic test performance. The positive predictive values were rather low for the Rydel Seiffer tuning fork, suggesting that those who had abnormal findings during clinical point-of-care testing were only a subset of those with neurosensory deficits according to vibrotactile QST. However, the positive predictive value for temperature rollers, especially in relation to CDT, was substantially higher. This difference was also contrasted in the negative predictive values, where the tuning fork was a more effective tool for identifying those with normal neurosensory function, which was not the case for the temperature rollers. The positive likelihood ratios ranged from 2.5 to 10.0, and the negative likelihood ratios from 0.4 to 0.8. A positive likelihood ratio above 10 indicates strong evidence to rule in injury whereas a negative likelihood ratio below 0.1 strongly rules out injury.^25^ Thus, the clinical point-of-care tools could not be considered to strongly rule in or out an injury. Finally, the diagnostic ORs ranged from 2.5 to 24.2. A value of 1 indicates that a certain test does not discriminate between injured and uninjured whereas higher values indicate a better discriminatory test performance.^26^ Our interpretation of the diagnostic ORs in our study was that clinical point-of-care testing did not discriminate sufficiently between those with normal or abnormal findings according to QST.

One interesting finding in our current study was that both clinical point-of-care and QST showed poorer perception for the little fingers compared with index fingers. The same pattern was found by Ekman et al,^7^ who reported that vibrotactile thresholds differed between index and little fingers, with correlation coefficients ranging from 0.44 to 0.61. In addition, Burström et al^19^ reported that thermal QST thresholds were wider apart for the little fingers compared with the index fingers. This notion should be considered when analyzing the results of neurosensory testing among HAV-exposed workers. HAV injury usually has an asymmetrical and patchy distribution without relation to dermatomes, and slightly poorer sensibility in the little fingers should be interpreted cautiously since this may indeed be normal. However, the transmissibility of HAV exposure differs between the fingers,^27^ and it is possible that the little finger with smaller mass is more vulnerable to mechanical injury than the index finger. We also found a higher proportion of abnormal findings for thermal compared with vibrotactile QST, indicating that small fiber neuropathy might be the first sign of incipient HAV injury and that thermal QST is a more effective method for early detection of such injury; this is in line with previous research.^20^

### 4.3. Methodological considerations

We used a convenience sampling method to recruit study participants from workplaces. This means that we do not know how many opted out or for what reason. However, our experience was that most workers were enthusiastic to participate to get an idea of whether they had signs of HAV injury or not. Thus, we do not believe that this strategy resulted in any major recruitment bias. However, symptomatic workers may have been more motivated to participate. On the other hand, there might have been a healthy worker effect, where subjects who had already contracted HAV injury, or other disabling conditions, had left the working life or at least HAV-exposed tasks. Moreover, we also recruited study participants from the Occupational and Environmental Medicine Clinic in the same region where the companies were located. These subjects had slightly more abnormal findings during clinical point-of-care testing and QST compared with the company-recruited subjects (data not shown). We chose this approach to get the full span of neurosensory function, from asymptomatic and uninjured subjects to those with established severe HAV injury. The participants worked in many different trades with varying types of handheld vibrating tools. We believe that our sample could be considered representative of what an occupational physician would encounter in everyday practice and that our results are generalizable to the OHS setting. However, since the prevalence of injury affects the diagnostic performance of the different tests, especially the positive and negative predictive values, some caution is warranted when interpreting our results. Despite efforts to include both men and women, there were very few female participants. This reflects the current situation in Sweden, where most HAV-exposed occupations are highly male-dominated, and this limits any conclusions regarding sex. We recruited workers from different occupations, where HAV exposure likely differed in both characteristics and intensity. For instance, mechanics used impact tools with transients that can be especially harmful, but our analyses were not stratified based on occupation or more detailed HAV exposure assessment. Another methodological consideration is the effect of aging. We did not adjust our analyses for age since we argue that both clinical point-of-care testing and QST would be similarly affected by the detrimental effects of aging. Also, since age was strongly correlated with HAV exposure, adjusting for age would have attenuated the effects of HAV exposure. Still, we could demonstrate that VPT increased with the duration of exposure (Figure 1), although the *z*_avg_ is based on age-matched reference values. Finally, the study was conducted in Sweden, and the findings may not be directly applicable to workers in other countries with different occupational structures.

There are also several strengths to our study. To begin with, we collected thorough data on previous injuries and diseases, medication, and family history to investigate other potential causes of peripheral sensory neuropathy. All study participants were then examined in a systematic manner using detailed protocols for each test that followed the national guidelines.^5^ The QST adhered to the standard protocols used at Swedish hospitals and by OHS providers, and evaluated both small and large fiber function (ie, vibrotactile and thermal perception thresholds). The number of examiners performing clinical point-of-care testing and QST was limited to reduce inter-rater variability, since especially the clinical point-of-care testing may be dependent on the examiner’s technique and experience.

## 5. Conclusions and implications

We conclude that performing vibrotactile and thermal QST could increase the detection rate of neurosensory injury among HAV-exposed workers. Since manifest injury cannot be treated, early detection is of utmost importance and QST should be implemented on a broader basis. Currently, QST is mostly performed at the Swedish university hospitals, and the feasibility of introducing QST on a large scale among OHS providers can be questioned. Since QST is expensive, time-consuming, and requires trained personnel, it is important to select appropriate groups for screening. The benefit of QST among HAV-exposed workers would likely be largest in the first decade of exposure, where subtle changes could be detected with QST before any clinical signs become apparent, and among workers with especially high exposure to HAV. Currently, vibrotactile QST is less expensive than thermal QST and could more easily be made available to more OHS providers. However, thermal QST offers the opportunity to investigate small fiber neuropathy, which appears to be the most frequent impairment among HAV-exposed workers and would also be beneficial to introduce to the screening procedure.

## Supplementary Material

Web_Material_uiaf034

## Data Availability

The dataset used in our study cannot be shared publicly due to legal and ethical reasons. We have collected personal data related to health among human subjects, which is protected by both national and EU legislation. Neither does the ethical approval allow us to publicly share the dataset. However, anonymized excerpts from the dataset can be made available upon reasonable request to the corresponding author.
