# Supplementary material for: The diagnostic test performance of clinical point-of-care testing in relation to quantitative sensory testing for neurosensory injury among workers exposed to hand-arm vibration
Source: J Occup Health. 2025 Jun 13;67(1):uiaf034. doi: 10.1093/joccuh/uiaf034 (PMC12234121; doi:10.1093/joccuh/uiaf034)
Supplement: Web_Material_uiaf034 [file web_material_uiaf034.zip › Supplementary material 1.pdf]

Article title: The diagnostic test performance of clinical point-of-care testing in relation to quantitative sensory testing for neurosensory injury among workers exposed to hand-arm vibration

Journal name: Journal of Occupational Health

Author names: Albin Stjernbrandt, Ingrid Liljelind, Eva Tekavec, Hans Pettersson

Affiliation and e-mail address of the corresponding author: Department of Epidemiology and Global Health, Umeå University, Umeå, Sweden, albin.stjernbrandt@umu.se

Supplementary table. Sensitivity analyses of the diagnostic test performance of the Rydel Seiffer tuning fork and temperature rollers, using vibrotactile and thermal quantitative sensory testing as reference methods but with a wider definition of normality.

Clinical test	Anatomical location	Abnormal finding	Quantitative sensory testing	Abnormal finding	Sensitivity (%)	Specificity (%)	Positive predictive value (%)	Negative predictive value (%)	Positive likelihood ratio	Negative likelihood ratio	Diagnostic odds ratio
		N (%)		N (%)	Estimate (95% CI)	Estimate (95% CI)	Estimate (95% CI)	Estimate (95% CI)	Estimate (95% CI)	Estimate (95% CI)	Estimate
Rydel Seiffer tuning fork	Right index finger	16 (8.1)	Vibration perception threshold (Z_{avg}) >2.0	16 (7.1)	45.5 (21.3–72.0)	94.1 (89.7–96.7)	31.3 (14.2–55.6)	96.7 (93.0–98.5)	7.7 (3.2–18.3)	0.6 (0.4–1.0)	13.3
	Right little finger	33 (16.8)	Vibration perception threshold (Z_{avg}) >2.0	26 (11.6)	50.0 (29.9–70.1)	87.0 (81.3–91.2)	30.3 (17.4–47.3)	93.9 (89.1–96.7)	3.9 (2.2–6.9)	0.6 (0.4–0.9)	6.7
	Left index finger	22 (11.1)	Vibration perception threshold (Z_{avg}) >2.0	14 (6.2)	63.6 (35.4–84.8)	92.0 (87.2–95.1)	31.8 (16.4–52.7)	97.7 (94.3–99.1)	8.0 (4.1–15.3)	0.4 (0.2–0.9)	20.1
	Left little finger	27 (13.7)	Vibration perception threshold (Z_{avg}) >2.0	22 (9.8)	52.9 (31.0–73.8)	90.0 (84.7–93.6)	33.3 (18.6–52.2)	95.3 (91.0–97.6)	5.3 (2.8–9.9)	0.5 (0.3–0.9)	10.1
Temperature rollers	Right index finger	44 (20.1)	Warm detection threshold (>42.0°C)	19 (11.4)	44.4 (24.6–66.3)	83.9 (77.0–89.0)	25.8 (13.7–43.2)	92.3 (86.4–95.8)	2.8 (1.5–5.2)	0.7 (0.4–1.0)	4.2
			Cold detection threshold (<23.0°C)	13 (7.8)	58.3 (32.0–80.7)	83.9 (77.2–88.9)	22.6 (11.4–39.8)	96.2 (91.3–98.3)	3.6 (2.0–6.6)	0.5 (0.3–1.0)	7.2
	Right little finger	73 (34.3)	Warm detection threshold (>42.0°C)	33 (19.9)	64.5 (46.9–78.9)	74.0 (65.8–80.9)	37.7 (25.9–51.2)	89.5 (82.2–94.0)	2.5 (1.7–3.7)	0.5 (0.3–0.8)	5.2
			Cold detection threshold (<23.0°C)	28 (16.9)	53.8 (35.5–71.2)	70.5 (62.2–77.6)	26.4 (16.4–39.6)	88.6 (81.1–93.3)	1.8 (1.17–2.84)	0.7 (0.43–1.01)	2.8
	Left index finger	40 (18.3)	Warm detection threshold (>42.0°C)	23 (13.9)	36.4 (19.7–57.0)	82.0 (74.8–87.5)	24.2 (12.8–41.0)	89.1 (82.5–93.4)	2.0 (1.05–3.90)	0.8 (0.56–1.07)	2.6
			Cold detection threshold (<23.0°C)	16 (9.7)	53.3 (30.1–75.2)	82.9 (75.9–88.1)	24.2 (12.8–41.0)	94.5 (89.1–97.3)	3.1 (1.72–5.64)	0.6 (0.33–0.97)	5.6
	Left little finger	65 (29.5)	Warm detection threshold (>42.0°C)	37 (22.6)	36.1 (22.5–52.4)	75.6 (67.3–82.3)	30.2 (18.6–45.1)	80.2 (72.0–86.4)	1.5 (0.87–2.53)	0.9 (0.65–1.10)	1.7
			Cold detection threshold (<23.0°C)	33 (20.0)	51.6 (34.8–68.0)	78.3 (70.4–84.5)	36.4 (23.8–51.1)	87.1 (79.8–92.0)	2.4 (1.48–3.82)	0.6 (0.43–0.90)	3.8
